# Traumatic Cystic Artery Pseudoaneurysm After Transjugular Liver Biopsy: A Case Report

**DOI:** 10.7759/cureus.75116

**Published:** 2024-12-04

**Authors:** Amy Bezold, Xuan Tran, Gautam Edhayan, Uzair Javaid, Alexander Shestopalov

**Affiliations:** 1 Vascular and Interventional Radiology, Oregon Health and Science University, Portland, USA; 2 Radiology, University of Texas Medical Branch, Galveston, USA; 3 Vascular and Interventional Radiology, University of Texas Medical Branch, Galveston, USA; 4 Interventional Radiology, Houston Vascular Care, Houston, USA

**Keywords:** coagulopathy, cystic artery pseudoaneurysm, endovascular embolization, hemobilia, transjugular liver biopsy

## Abstract

Cystic artery pseudoaneurysms are a rare but life-threatening entity that commonly occurs as a sequela to acute cholecystitis. We present a case of a 52-year-old male with a past medical history of decompensated alcoholic liver cirrhosis who underwent a transjugular liver biopsy (TJLB) after correction of his baseline coagulopathy. On post-operative day one, the patient had significant blood loss with an inappropriate response to blood transfusions and without an identifiable source of bleeding. Imaging was obtained, which revealed findings for concerning active hemorrhage into the gallbladder lumen. Angiography was performed, demonstrating a cystic artery pseudoaneurysm with resultant bleeding into the gallbladder, necessitating intervention. The patient underwent successful endovascular embolization of the superior and inferior divisions of the distal cystic artery using multiple metallic coils. The patient was discharged after six days post-operatively without further incident. Follow-up imaging demonstrated no residual filling of the cystic artery pseudoaneurysm. This case highlights an important, albeit rare, complication of TJLB that interventionalists should be aware of due to its rarity and the potential for significant morbidity.

## Introduction

Cystic artery pseudoaneurysms are a rare entity causing hemodynamically significant and life-threatening hemorrhages that are usually secondary to acute cholecystitis [1]. Other etiologies for the development of cystic artery pseudoaneurysms are pancreatitis, cholelithiasis, and trauma during cholecystectomy [1]. The incidence of vascular injury during cholecystectomy is low (0.2-0.5%) and pseudoaneurysms associated with laparoscopic cholecystectomy generally arise from the right hepatic artery. The pathogenesis of cystic or hepatic artery pseudoaneurysms is thought to be related to inflammatory damage and weakening of the adventitia caused by cholecystitis or pancreatitis, which may be exacerbated by manipulation, clip application, or thermal injury [1]. Complications of cystic artery pseudoaneurysms are serious and can include bleeding into the biliary system or peritoneum necessitating resuscitation and requiring prompt intervention [1]. We present a case of an iatrogenic cystic artery pseudoaneurysm arising as a complication of transjugular liver biopsy (TJLB).

TJLB is a widely performed and useful technique in patients with severe coagulopathy and/or severe ascites [2]. TJLB is performed via a needle introduced through a hepatic vein, without causing a transcapsular injury, so any bleeding related to the procedure will drain back into the venous system. Since it was initially described in 1964, many large studies have shown it is a safe technique with complications reported between 0.6% and 20%, the most common major complications being intraperitoneal bleeding, vessel injury, or ventricular arrhythmia [2-4]. In the present case, due to patient-specific anatomy, the biopsies were taken from an accessory hepatic vein resulting in a traumatic cystic artery pseudoaneurysm.

## Case presentation

A 52-year-old male was originally admitted with complaints of abdominal pain and chills. His past medical history includes alcoholic liver cirrhosis (model for end-stage liver disease {MELD}-Na 30, Child-Pugh Class C). He was being evaluated for liver transplantation. Interventional radiology was consulted for transjugular hepatic pressure measurements and liver biopsy.

Pre-procedural lab work showed an international normalized ratio (INR) of 3.6, fibrinogen of 81 mg/dL, 67 platelets/µL, and hemoglobin of 11.6 g/dL. After the transfusion of two units of fresh frozen plasma and cryoprecipitate, the INR and fibrinogen improved to 2.7 and 152 mg/dL, respectively.

**Figure 1:**
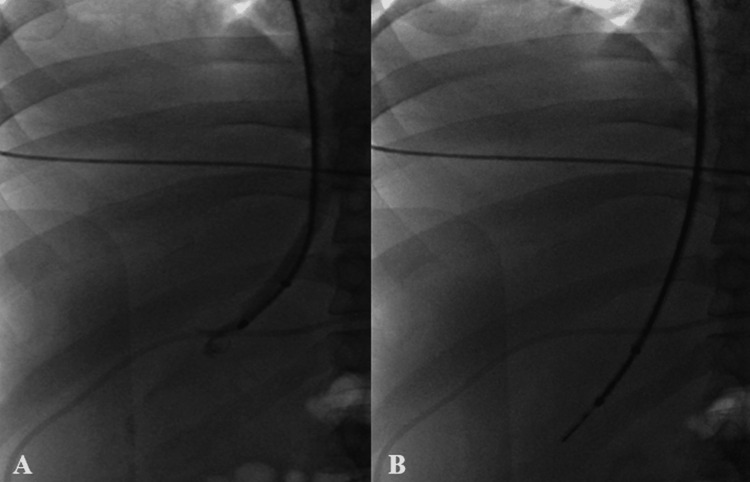
Hepatic venogram and biopsy needle deployment. (A) Selective free hepatic venogram showing the multipurpose angled sheath within an accessory hepatic vein prior to biopsy or pressure measurements. (B) Deployment of a Quick-Core Biopsy Needle (Bloomington, IN: Cook Medical) within the accessory hepatic vein.

At the start of the procedure, the right internal jugular vein was accessed under ultrasound guidance utilizing a micropuncture system (Bloomington, IN: Cook Medical), and a 10-french Check-Flo sheath (Bloomington, IN: Cook Medical) was placed. Through the sheath, a multipurpose angled catheter (Shibuya, Japan: Terumo) was used to access a hepatic vein. Multiple attempts were made to access the main right hepatic vein; however, this was not possible due to sharply angulated anatomy at the venous origin. Selective free hepatic venography was performed showing the catheter within an accessory right hepatic vein (Figure 1A). At this point, a Fogarty balloon catheter (Irvine, CA: Edwards Lifesciences) was advanced into the accessory hepatic vein, and pressure recordings were obtained with a portosystemic gradient of 20 mmHg. Next, the Rösch-Uchida transjugular liver access set and Quick-Core Biopsy Needle (Bloomington, IN: Cook Medical) were placed within the accessory hepatic vein and two core needle biopsies were then taken through the system (Figure 1B). Multiple venograms throughout the procedure, before and after the biopsy, showed that the sheath was persistently within the hepatic vein. After the removal of the sheaths, hemostasis was obtained at the internal jugular vein using manual compression.

**Figure 2:**
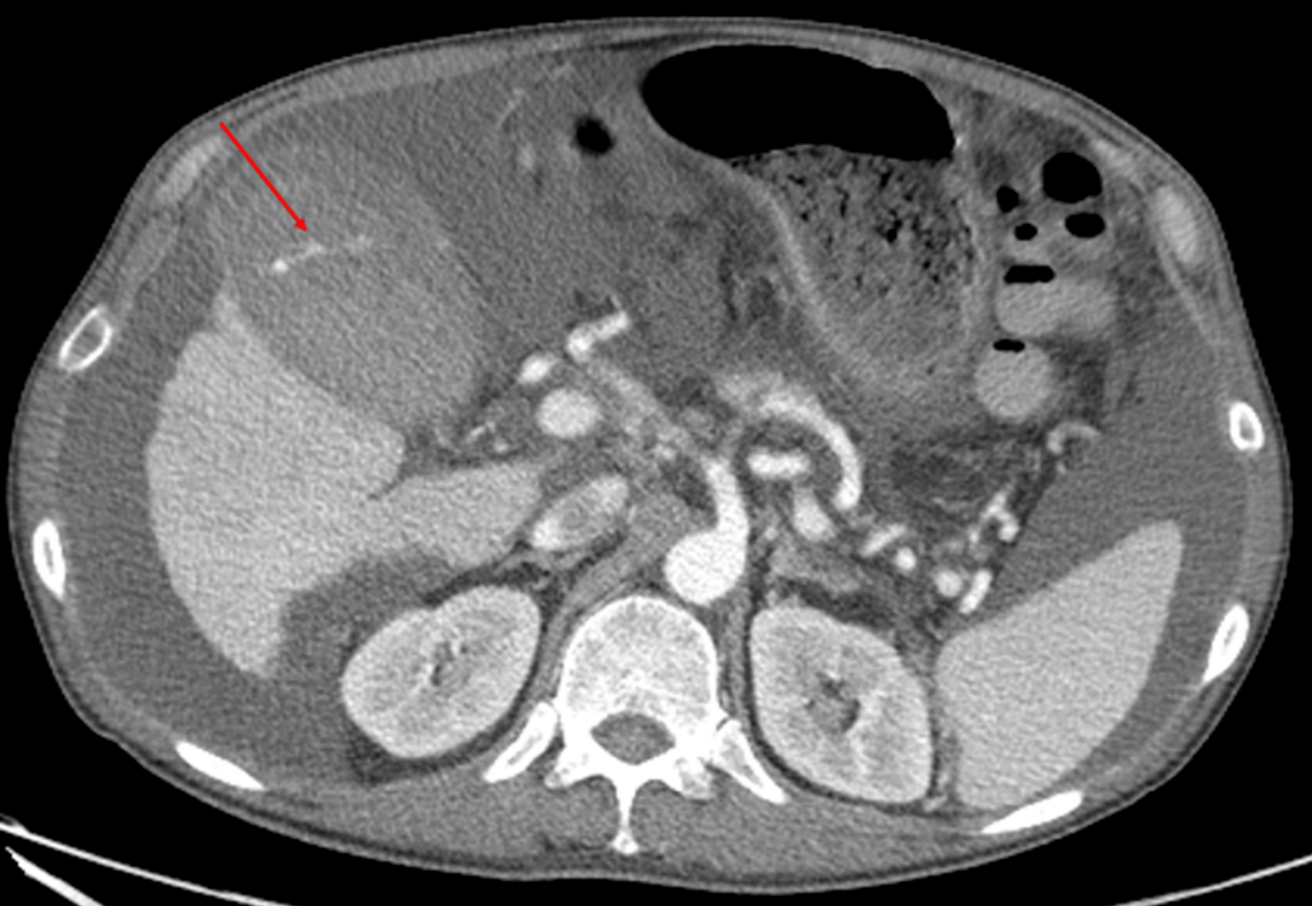
Axial computed tomography (CT) imaging with intravenous contrast in the arterial phase. Imaging obtained after the patient developed abdominal pain and acute anemia post-transjugular liver biopsy shows active contrast extravasation into the gallbladder lumen (red arrow).

Post-procedurally, the patient’s hemoglobin steadily trended down from pre-procedural values of 11.6 g/dL to 6.4 g/dL despite transfusion of multiple units of packed red blood cells. Multiphasic computed tomography angiogram of the abdomen was performed which showed arterial phase contrast extravasation into the gallbladder lumen with pooling of contrast in the lumen on delayed phases (Figure 2). The patient was then taken back to the fluoroscopy suite for a mesenteric angiogram with possible embolization.

**Figure 3:**
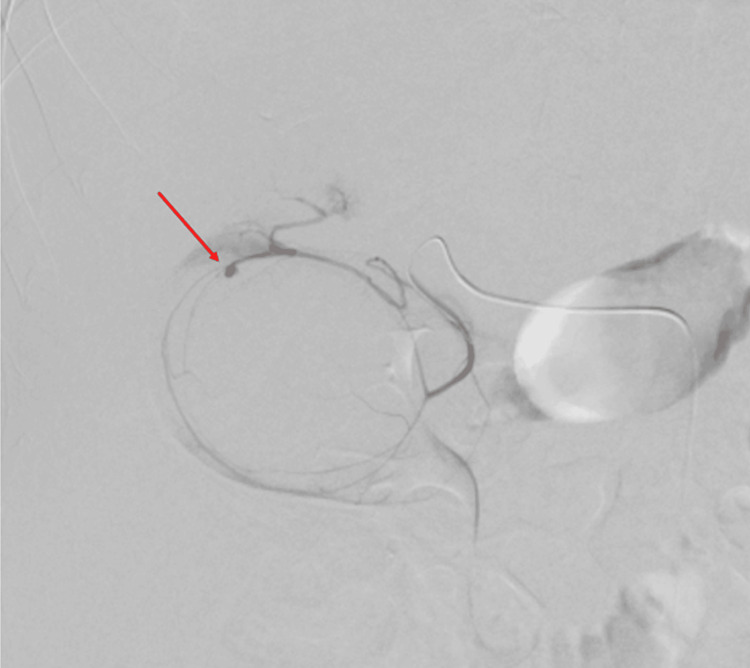
Superselective angiogram from the proximal cystic artery. Irregular traumatized vessels and a 3 x 3 mm focal, rounded contrast extravasation at the superior aspect of the gallbladder, consistent with a pseudoaneurysm (red arrow), are demonstrated.

The micropuncture kit was used to access the right common femoral artery and a 6-french access sheath (Shibuya, Japan: Terumo) was placed. The celiac and superior mesenteric arteries were catheterized using a C2 catheter (Bloomington, IN: Cook Medical). Angiograms from the celiac artery showed a faint vascular blush in the gallbladder. The proximal gastroduodenal artery was accessed through a microcatheter system using a Progreat microcatheter (Shibuya, Japan: Terumo) and Fathom microwire (Marlborough, MA: Boston Scientific). Superselective angiograms from the proximal cystic artery showed a 3 x 3 mm pseudoaneurysm with contrast blush into the gallbladder lumen arising from the distal superior division of the cystic artery (Figure 3). Irregular traumatized vessels were also seen at the superior aspect of the gallbladder.

**Figure 4:**
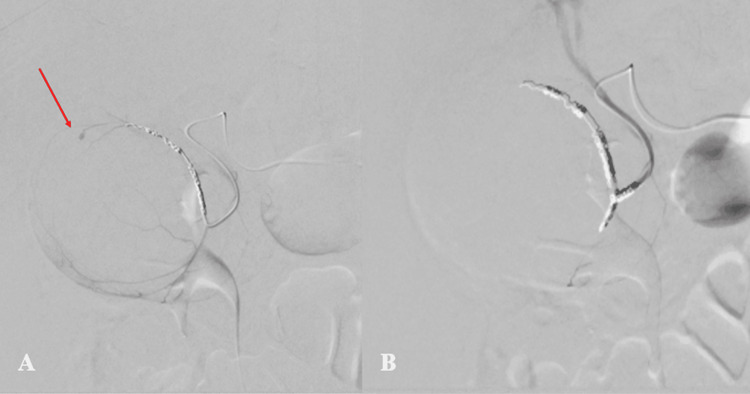
Initial and final post-embolization angiograms. (A) Initial post-embolization angiogram from the mid-cystic artery shows embolization coils in the distal cystic artery superior division with continued filling of the pseudoaneurysm from the inferior division of the cystic artery (red arrow). (B) Final post-embolization angiography from the mid-cystic artery demonstrates metallic coils in the distal superior and inferior divisions of the cystic artery with no filling of the previously seen pseudoaneurysm and no active contrast extravasation.

The microsystem was advanced to the superior division of the distal cystic artery, and embolization was performed using Nester metallic coils (Bloomington, IN: Cook Medical). Post-embolization angiogram from the cystic artery demonstrated continued filling of the pseudoaneurysm from the inferior division of the cystic artery (Figure 4A). The inferior division was similarly embolized with metallic coils with post-embolization angiography from the cystic artery demonstrating no filling of the previously seen pseudoaneurysm as well as no active extravasation (Figure 4B).

Hemostasis of the right common femoral arteriotomy site was achieved using a StarClose SE closure device (Chicago, IL: Abbott Laboratories). The patient tolerated the procedure well and required no further blood products during admission and was discharged on post-operative day six. The patient has been listed on the liver transplant waiting list at this time and is awaiting transplantation. He has undergone a repeat multiphasic computed tomography angiogram for hepatocellular carcinoma screening without evidence of pseudoaneurysm recurrence, non-target embolization, or complication secondary to endovascular embolization.

## Discussion

Cystic artery pseudoaneurysms are a rare entity that generally arise from complications associated with acute cholecystitis. We present a case of an iatrogenic cystic artery pseudoaneurysm after TJLB with biopsy samples taken from an accessory right hepatic vein. Given the unfavorable right main hepatic vein anatomy in this patient, TJLB through the accessory vein was thought to be less risky for the patient than conversion to a percutaneous approach. Sampling from the accessory right hepatic vein is an accepted approach; however, it has been reported to be associated with an increased risk of complications, including direct arterial injury, liver capsule perforation, or even accidental renal sampling [5,6]. In the present case, biopsies taken from this position likely caused trauma to the superior division of the distal cystic artery and resultant hemorrhage and hemobilia.

Transarterial embolization has been shown to have an approximately 95% success rate in treating post-laparoscopic cholecystectomy cystic and hepatic artery pseudoaneurysms, with less morbidity and mortality than repeat surgery [7]. However, approximately 88% of pseudoaneurysms associated with laparoscopic cholecystectomy arise from the right hepatic artery. Only a few cases have been reported to be secondary to TJLB, and of these cases, the traumatized artery is a branch of the hepatic artery [8]. The present literature review did not identify the case of cystic artery pseudoaneurysm after TJLB.

## Conclusions

Cystic artery pseudoaneurysms are rare complications usually associated with acute cholecystitis and laparoscopic cholecystectomy. We discussed a rare case of iatrogenic cystic artery pseudoaneurysm after TJLB. This rare yet life-threatening complication impacts patient outcomes. This case raises the idea that close post-procedure monitoring for hemodynamic instability or changes in laboratory parameters after TJLB may be warranted in patients with risk factors for hemorrhage, such as baseline coagulopathy to rule out rare yet morbid complications such as iatrogenic cystic artery pseudoaneurysm.
